# Selection of promising accessions of phalsa (*Grewia asiatica* L.) based on fruit‐related traits

**DOI:** 10.1002/fsn3.2967

**Published:** 2022-07-18

**Authors:** Farhad Mirheidari, Ali Khadivi, Younes Moradi

**Affiliations:** ^1^ Department of Horticultural Sciences, Faculty of Agriculture and Natural Resources Arak University Arak Iran

**Keywords:** breeding, cultivation, fruit quality, germplasm, *Grewia asiatica*

## Abstract

Phalsa or falsa (*Grewia asiatica* L., family Malvaceae) is a promising, yet underutilized berry fruit of tropical regions. It contains a rich source of various bioactive compounds, such as anthocyanins, tannins, phenols, and flavonoids. In the present study, morphological and pomological diversity of 48 accessions of this species was evaluated to introduce superior selections. Considerable variability was detected among the accessions studied based on the characteristics recorded. Fruit shape exhibited the highest CV (69.66%), while seed length showed the lowest CV (7.98%). Fruit color showed strong diversity, including red, red‐purple, purple‐cream, purple, and dark purple. Fruit weight ranged from 0.29 to1.14 g, and fruit flesh thickness varied from 1.90 to 3.91 mm. Principal component analysis (PCA) showed that 82.64% of the variability observed was explained by the first 13 components. A dendrogram created using cluster analysis grouped the accessions into two major clusters. Based on the traits related to fruit quality, such as fruit weight, fruit color, fruit flesh color, and fruit taste, 14 accessions, including Talsar‐6, Talsar‐8, Ganjabad‐31, Talsar‐4, Ganjabad‐18, Ganjabad‐24, Talsar‐5, Ganjabad‐25, Ganjabad‐30, Ganjabad‐17, Talsar‐7, Talsar‐3, Talsar‐2, and Talsar‐1, were superior. It is recommended to use the best accessions selected in breeding programs.

## Graphical abstract

Principal component analysis (PCA) showed that 82.64% of the variability observed was explained by the first 13 components. The dendrogram created using cluster analysis grouped the accessions into two major clusters.

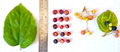

## 1 INTRODUCTION

Phalsa or falsa (*Grewia asiatica* L., family Malvaceae) is a promising, yet underutilized berry fruit of tropical regions. It is a 4 to 5 m tall shrub. The leaves are approximately 5–18 cm long and broad. The flowers are arranged in cymes of several together: the individual flowers are yellowish in color with five large (12 mm) sepals and five smaller (4–5 mm) petals. The flower has a diameter of about 2 cm (Dave et al., 2016).

Phalsa contains a rich source of various bioactive compounds, such as anthocyanins, tannins, phenols, and flavonoids (Dave et al., 2016; Wani et al., 2015). Further, its fruit pulp contains flavonoids, proteins, and amino acids (Lakshmi et al., 1976) that are a good source of nutrition. Also, other parts of this plant have been used as herbal medicine for the treatment of various diseases, such as cancer, aging, fever, rheumatism, and diabetes (Ullah et al., 2012; Siddiqi et al., 2011).

Phalsa has tremendous commercial utilization. Ripe phalsa fruits are eaten and made into soft drinks and squash in India during summer. Leaves are utilized as animal food, bark as substitute cleanser in Burma, and adhesive concentrate of bark is utilized in sugar. Fiber acquired from the bark is utilized to make twines (Paul, 2015). Wood can be used for making bows, shingles, and shafts for conveying loads on shoulders (Yadav, 1999).

Characterization of crop genetic resources is an essential step to develop conservation and breeding strategies in crop improvement programs. Germplasm characterization usually comprise of a detailed description of accessions for morphological and agronomic traits, as well as other useful attributes. Quantitative and qualitative phenotypic traits aid in identifying differences between individuals of a species (Achigan‐Dako et al., 2015). Quantitative traits are measurable characteristics and are generally controlled by multiple genes and highly influenced by the environment. Conversely, qualitative traits have categorical values and are categorized as binary or multi‐categorical (defined by several categories or classes), and are controlled by single dominant or recessive genes and less influenced by the environment (Szamosi et al., 2009). Morphological characteristics are prerequisite for any food product and provide useful information regarding designing and development of equipment used during various unit operations, such as handling, transportation, sorting, separating, packing, and processing of fruits (Yildiz et al., 2015). The phenotypic diversity of *G. asiatica* has not been investigated in Iran yet. Therefore, the objective of the present study was to assess the phenotypic diversity of this species to select desirable accessions with suitable agronomic and horticultural traits for direct production, breeding, and conservation.

## 2 MATERIAL AND METHODS

### 2.1 Plant material

The morphological and pomological diversity of 48 accessions of *G. asiatica* was evaluated to introduce superior selections from two areas of Sistan‐va‐Baluchestan province, Iran, including Ganjabad and Talsar. The Ganjabad area is located at 26°25′45“N latitude, 61°17′13”E longitude, and 595 m height above sea level. The Talsar area is located at 27°07′36“N latitude, 61°36′02”E longitude, and 285 m height above sea level. The accessions were named based on their area.

### 2.2 The characters evaluated

Forty‐four morphological and pomological traits were used to evaluate phenotypic diversity and select superior accessions (Table 1). A total of 50 adult leaves and 50 mature fruits per accession were randomly selected and harvested. The traits related to dimensions of leaf, fruit, and seed were measured using a digital caliper. A digital scale with an accuracy of 0.01 g was used to measure the weight of fruit and seed. The qualitative traits (Table 2) were visually examined and coded.

**TABLE 1:** Statistical descriptive parameters for morphological traits used to study *G. asiatica* accessions

No.	Trait	Unit	Min.	Max.	Mean	SD	CV (%)
1	Tree growth habit	Code	1	5	2.54	1.50	59.09
2	Tree growth vigor	Code	1	5	3.62	1.44	39.72
3	Tree height	Code	1	5	2.46	1.22	49.59
4	Branching	Code	1	5	2.38	1.44	60.42
5	Branch density	Code	1	5	3.17	1.48	46.62
6	Branch flexibility	Code	1	5	4.00	1.31	32.63
7	Trunk type	Code	1	5	2.62	1.53	58.21
8	Trunk diameter	Code	1	5	3.17	1.29	40.82
9	Trunk color	Code	1	7	4.00	1.60	39.95
10	Canopy density	Code	1	5	2.83	1.00	35.19
11	Tendency to form suckers	Code	1	5	4.00	1.31	32.63
12	Leaf density	Code	1	5	4.12	1.08	26.31
13	Leaf shape	Code	1	3	1.46	0.85	58.15
14	Leaf apex shape	Code	1	3	1.33	0.75	56.62
15	Leaf base shape	Code	1	3	1.54	0.90	58.31
16	Leaf color	Code	1	3	2.04	1.01	49.51
17	Leaf serration depth	Code	1	5	3.79	1.22	32.19
18	Leaf length	mm	97.23	205.61	148.52	32.30	21.75
19	Leaf width	mm	84.22	156.87	118.77	21.10	17.77
20	Petiole length	mm	14.18	21.37	17.82	1.89	10.63
21	Petiole width	mm	1.65	2.95	2.28	0.32	13.99
22	Ripening date	Date	Late May	Early June	2.29	0.97	42.23
23	Fruit density	Code	1	5	4.33	1.12	25.80
24	Maximum of fruit number per node	Number	11	22	16.25	2.69	16.58
25	Fruit stalk length	mm	2.73	4.55	3.51	0.57	16.15
26	Fruit stalk diameter	mm	1.25	2.10	1.55	0.18	11.34
27	Fruit stalk length on bunch	mm	10.04	15.32	11.94	1.28	10.75
28	Fruit stalk diameter on bunch	mm	0.53	1.12	0.87	0.13	15.57
29	Fruit shape	Code	1	5	2.38	1.66	69.66
30	Fruit length	mm	7.99	12.35	10.56	0.93	8.79
31	Fruit width	mm	7.56	13.63	10.95	1.35	12.37
32	Fruit weight	g	0.29	1.14	0.81	0.24	29.62
33	Fruit color	Code	1	9	4.75	2.94	61.79
34	Fruit flesh color	Code	1	5	3.38	1.83	54.11
35	Fruit taste	Code	1	3	1.75	0.98	55.89
36	Fruit flesh firmness	Code	1	5	3.79	1.29	33.98
37	Fruit flesh thickness	mm	1.90	3.91	2.97	0.44	14.86
38	Fruit juice color	Code	1	5	3.21	1.38	43.08
39	Seed number per fruit	Number	1	3	1.69	0.69	40.77
40	General shape of seeds	Code	1	5	4.08	1.43	34.98
41	Seed shape	Code	1	7	4.88	2.08	42.62
42	Seed length	mm	4.71	6.51	5.68	0.45	7.98
43	Seed width	mm	3.94	6.09	4.94	0.51	10.22
44	Total weight of seeds	g	0.04	0.11	0.07	0.01	21.41

**TABLE 2:** Frequency distribution for the measured qualitative morphological characters in the studied *G. asiatica* accessions

	Frequency (no. of accessions)				
Trait	1	3	5	7	9
Tree growth habit	Weeping (20)	Spreading (19)	Open (9)	—	—
Tree growth vigor	Low (7)	Moderate (19)	High (22)	—	—
Tree height	Low (17)	Moderate (27)	High (4)	—	—
Branching	Low (22)	Moderate (19)	High (7)	—	—
Branch density	Low (11)	Moderate (22)	High (15)	—	—
Branch flexibility	Low (4)	Moderate (16)	High (28)	—	—
Trunk type	Multi‐trunk/Low (19)	Multi‐trunk/Moderate (19)	Multi‐trunk/High (10)	—	—
Trunk diameter	Low (8)	Moderate (28)	High (12)	—	—
Trunk color	Brown (6)	Gray‐white (15)	Gray (24)	Dark gray (3)	—
Canopy density	Low (8)	Moderate (36)	High (4)	—	—
Tendency to form suckers	Low (4)	Moderate (16)	High (28)	—	—
Leaf density	Low (1)	Moderate (19)	High (28)	—	—
Leaf shape	Cordate (37)	Ovate (11)	—	—	—
Leaf apex shape	Acute (40)	Cuspidate (8)	—	—	—
Leaf base shape	Cordate (35)	Rounded (13)	—	—	—
Leaf color	Light green (23)	Green (25)	—	—	—
Leaf serration depth	Low (3)	Moderate (23)	High (22)	—	—
Ripening date	Late May (17)	Early June (31)	—	—	—
Fruit density	Low (2)	Moderate (12)	High (34)	—	—
Fruit shape	Oblate (26)	Round (11)	Ellipsoid (11)	—	—
Fruit color	Red (15)	Red‐purple (4)	Purple‐cream (6)	Purple (18)	Dark purple (5)
Fruit flesh color	White‐green (16)	Cream (7)	Pink (25)	—	—
Fruit taste	Sour (30)	Sour–sweet (18)	—	—	—
Fruit flesh firmness	Low (4)	Moderate (21)	High (23)	—	—
Fruit juice color	Colorless (9)	Pink (25)	Light red (14)	—	—
General shape of seeds	Globose (6)	Ellipsoid (10)	Ovate (32)	—	—
Seed shape	Ellipsoid (3)	Ovate (18)	One‐third sphere (6)	Hemisphere (21)	—

### 2.3 Statistical analysis

Analysis of variance (ANOVA) was performed to evaluate the variation among accessions based on the traits measured using SAS software (SAS Institute, Cary, NC, USA, 1990). Simple correlations between traits were determined using Pearson correlation coefficients (SPSS Inc., Chicago, IL, USA, Norusis, 1998). Principal component analysis (PCA) was used to investigate the relationship between accessions and determine the main traits effective in accession segregation using SPSS software. Hierarchical cluster analysis (HCA) was performed using Ward's method and Euclidean coefficient using PAST software (Hammer et al., 2001). The first and second principal components (PC1/PC2) were used to create a scatter plot with PAST software.

## 3 RESULTS AND DISCUSSION

Considerable variability was detected among the accessions studied based on the characteristics recorded. Fruit shape exhibited the highest CV (69.66%), while seed length showed the lowest CV (7.98%). The CV in 31 of 44 characters measured was more than 20.00%, indicating strong diversity among the accessions (Table 1).

Three types of tree growth habit were observed, including weeping (20 accessions), spreading (19), and open (9). Tree growth vigor was low (7 accessions), moderate (19), and high (22). Tree height and trunk diameter were predominantly moderate (27 and 28 accessions, respectively) (Table 2). Trunk color was highly variable, including brown (6), gray‐white (15), gray (24), and dark gray (3). Leaf shape was cordate in 37 and ovate in 11 accessions, leaf apex shape was acute (40) and cuspidate (8), while leaf base shape was cordate (35) and rounded (13). Leaf color was light green in 23 and green in 25 accessions, while leaf serration depth was low (3 accessions), moderate (23), and high (22). The range of leaf‐related characteristics was as follows: leaf length: 97.23–205.61 mm, leaf width: 84.22–156.87 mm, petiole length: 14.18–21.37 mm, and petiole width: 1.65–2.95 mm (Table 1).

Ripening date varied from late May (17 accessions) to Early June (31). Fruit density was predominantly high (34 accessions). Yield of the plant is one of the main reasons for the availability (Dhawan et al., 1993). Fruit shape was oblate in 26, round in 11, and ellipsoid in 11 accessions. Bala and Baramanray (2019a) reported the spherical fruit shape in tall and dwarf phalsa (*G. asiatica*) accessions. Fruit color showed strong diversity, including red (15), red‐purple (4), purple‐cream (6), purple (18), and dark purple (5). Bala and Baramanray (2019a) reported the dark purple fruit color for dwarf and yellowish‐purple for tall phalsa accessions from India, while Bala and Baramanray (2019b) reported dark purplish fruit color in a dwarf phalsa accession from India. Fruit flesh color was white‐green in 16, cream in 7, and pink in 25 accessions, while fruit juice color was colorless (9 accessions), pink (25), and light red (14) (Table 2). Bala and Baramanray (2019a) reported the greenish purple flesh color in a tall phalsa accession and attractive dark purple flesh color in a dwarf phalsa accession from India.

Sour fruit taste was predominant (30 accessions), while it was sour–sweet in 18 accessions. Bala and Baramanray (2019a) reported that fruits of a tall phalsa accession showed sweet taste and comparatively little astringency, whereas the fruit taste of a dwarf phalsa accession was found to be a mix of acid and sugar with high astringency. Fruit flesh firmness was low (4 accessions), moderate (21), and high (23). Bala and Baramanray (2019a) reported that fruits in a dwarf phalsa accession spoiled faster (more perishable nature) as compared with a tall accession (less perishable nature), but outer surface in fruits of a dwarf accession was found smooth, while fruits of a tall accession showed wrinkled and dried outer surface.

The maximum fruit number per node ranged from 11 to 22. The range of fruit stalk‐related characteristics was as follows: stalk length: 2.73–4.55 mm, stalk diameter: 1.25–2.10 mm, stalk length on bunch: 10.04–15.32 mm, and stalk diameter on bunch: 0.53–1.12 mm. The range of fruit‐related characteristics was as follows: fruit length: 7.99–12.35 mm, fruit width: 7.56–13.63 mm, fruit weight: 0.29–1.14 g, and fruit flesh thickness: 1.90–3.91 mm (Table 1). Bala and Baramanray (2019a) reported the average of 8.17 mm for fruit length and 8.30 mm for fruit width in a tall phalsa accession and also the average of 10.00 mm for fruit length and 10.22 mm for fruit width in a dwarf phalsa accession. Bala and Baramanray (2019b) reported the average of 10.00 mm for fruit length and 10.26 mm for fruit width in a dwarf phalsa accession.

**FIGURE 1:**
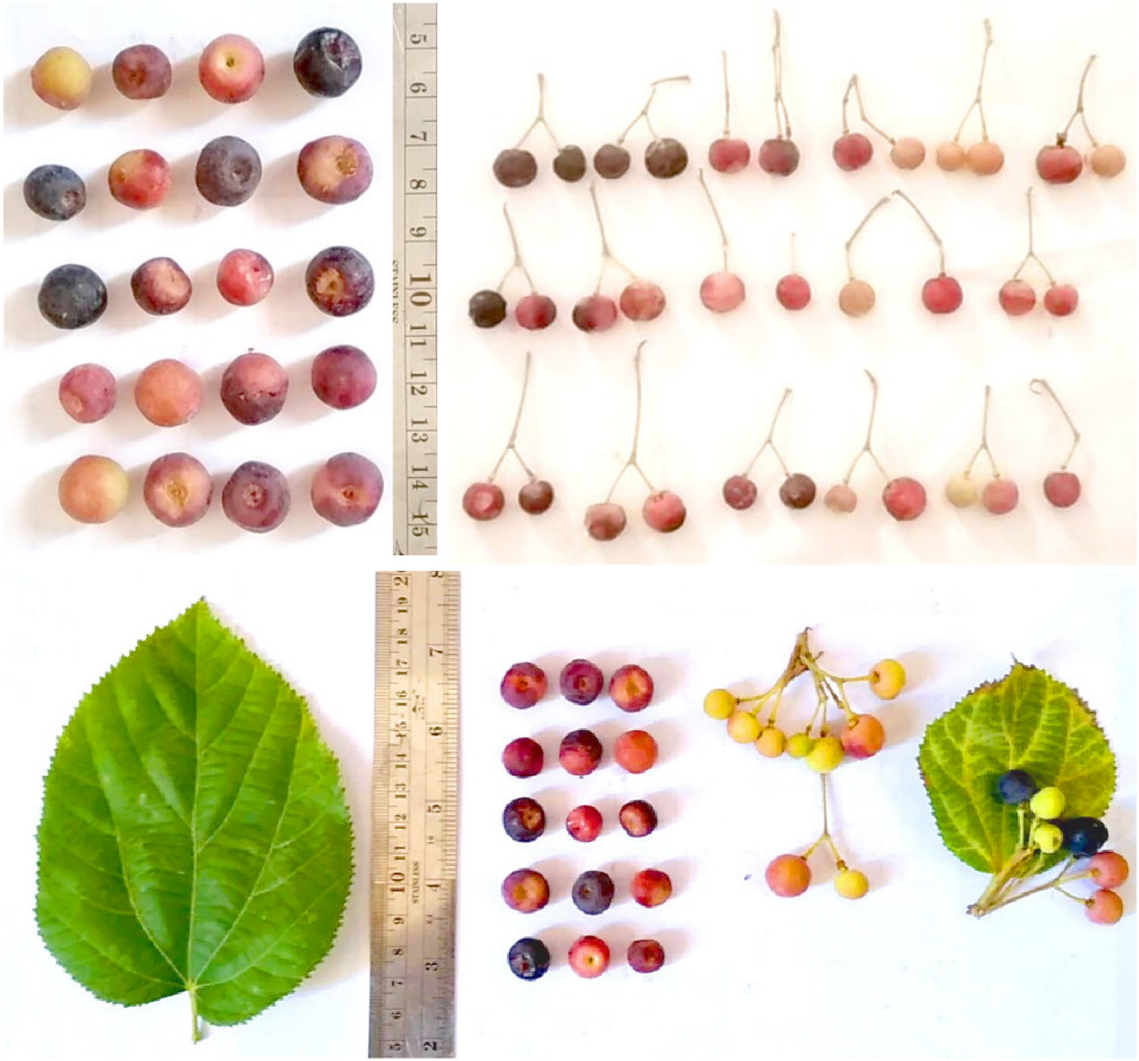
The pictures of leaves and fruits of *G. asiatica* accessions studied

Seed shape was highly variable and included ellipsoid (3 accessions), ovate (18), one‐third sphere (6), and hemisphere (21) (Table 2). Seed number per fruit was 1–3 (Table 1). Bala and Baramanray (2019b) reported the range of 1–2 seeds per fruit in a dwarf phalsa accession. The range of seed‐related characteristics was as follows: seed length: 4.71–6.51 mm, seed width: 3.94–6.09 mm, and total weight of seeds: 0.04–0.11 g (Table 1). Bala and Baramanray (2019a) reported comparatively large seed size in a tall and small seed size in a dwarf phalsa accession. The pictures of leaves and fruits of *G. asiatica* accessions studied are presented in Figure 1.

Correlation coefficients showed significant correlations between some characteristics recorded (data not shown). Leaf length showed positive and significant correlations with leaf width (*r* = .61) and petiole length (*r* = .52). Fruit weight showed positive and significant correlations with fruit length (*r* = .78), fruit width (*r* = .75), and fruit flesh thickness (*r* = .71). Fruit flesh firmness showed positive and significant correlations with seed length (*r* = .45), seed width (*r* = .44), and total weight of seeds (*r* = .48).

**FIGURE 2:**
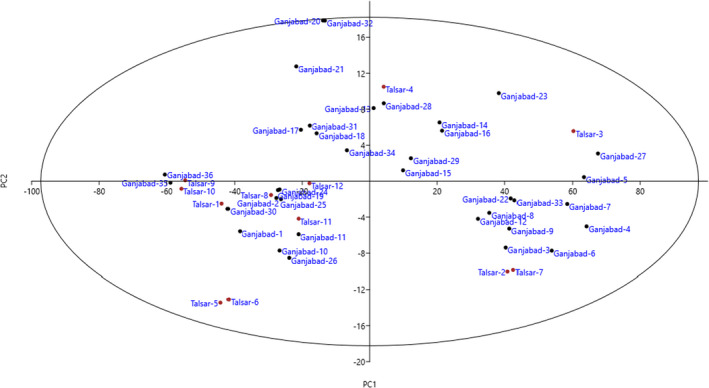
Scatter plot for the studied *G. asiatica* accessions based on PC1/PC2

The PCA was used to establish the relationships among the accessions and to analyze the correlations between phenotypic traits. The PCA used showed that 82.64% of the variability observed was explained by the first 13 components (Table 3). The PC1, PC2, and PC3 accounted for 11.04%, 8.64%, and 7.18% of the variability, respectively (26.86% in total). The PC1, which is the most important component, was correlated with fruit shape, fruit length, fruit width, fruit weight, fruit flesh thickness, and fruit juice color. Moreover, the characteristics with the greatest weight on PC2 were leaf length, leaf width, and petiole length. The PC3 was correlated with fruit flesh firmness, seed length, seed width, and total weight of seeds. The PCA provided a simplified classification of the accessions for collecting and breeding. The bi‐plot axes, created based on PC1 and PC2, which accounted for 19.68% of total variance, showed phenotypic distances among the accessions that reflected similarity and dissimilarity among them in terms of the variables measured (Figure 2). The accessions were distributed into four sides of the plot. The scatter plot showed that residuals of all the accessions bounce randomly around the 0.00 line, forming a horizontal band. This suggests that the variances of the error terms are equal and the relationship among the accessions is linear.

**TABLE 3:** Eigenvalues of the principal component axes from the PCA of the morphological characters in the studied *G. asiatica* accessions

	Component												
Trait	1	2	3	4	5	6	7	8	9	10	11	12	13
Tree growth habit	−0.11	0.08	0.13	0.27	−0.04	−0.10	0.76**	0.18	−0.06	−0.16	0.12	0.03	−0.06
Tree growth vigor	0.00	0.10	0.02	0.85**	−0.10	0.03	−0.03	0.09	−0.09	0.06	0.00	−0.11	0.07
Tree height	−0.06	0.01	0.21	0.66**	0.18	−0.05	0.45	−0.07	0.01	0.08	0.00	−0.09	−0.15
Branching	0.26	−0.17	0.05	0.31	0.04	−0.05	0.14	0.71**	0.11	−0.08	0.23	−0.07	0.06
Branch density	−0.33	0.33	−0.17	0.37	0.15	−0.06	−0.04	0.52**	0.24	0.20	0.13	−0.05	−0.16
Branch flexibility	−0.06	−0.10	0.22	0.04	−0.18	−0.08	−0.78**	−0.05	−0.10	0.35	0.06	0.07	−0.09
Trunk type	0.48	0.03	−0.01	0.25	−0.03	0.22	−0.13	0.26	0.43	−0.09	−0.04	0.36	−0.16
Trunk diameter	0.17	0.01	−0.05	0.29	−0.16	0.01	0.07	0.13	−0.08	0.14	−0.17	−0.73**	−0.04
Trunk color	−0.28	0.22	−0.26	−0.37	0.13	−0.03	−0.14	−0.29	−0.33	−0.10	0.23	−0.39	0.05
Canopy density	0.13	−0.12	0.40	0.26	−0.14	−0.04	−0.23	−0.03	−0.12	0.51	0.39	0.05	−0.21
Tendency to form suckers	0.16	0.19	−0.04	0.33	0.00	−0.20	−0.18	0.19	0.19	0.68**	−0.04	−0.10	0.22
Leaf density	−0.13	0.19	−0.20	0.68**	0.18	−0.01	0.02	0.03	0.14	0.07	0.27	−0.06	−0.31
Leaf shape	0.02	0.12	−0.01	−0.09	−0.04	0.85**	−0.07	−0.07	−0.25	−0.31	−0.09	−0.01	0.00
Leaf apex shape	0.01	−0.22	0.09	0.07	0.06	−0.16	−0.05	−0.13	0.11	−0.09	0.54**	0.38	0.39
Leaf base shape	−0.03	0.03	0.14	0.03	0.16	0.90**	0.05	−0.12	−0.03	−0.05	−0.05	0.10	0.04
Leaf color	0.34	−0.29	−0.28	0.11	−0.08	−0.10	0.08	0.00	0.02	0.12	0.22	0.41	−0.26
Leaf serration depth	0.05	0.00	−0.12	−0.12	−0.03	−0.04	0.02	−0.12	0.12	−0.07	0.15	0.02	0.85**
Leaf length	0.01	0.91**	0.11	0.17	−0.11	0.13	−0.01	−0.12	0.00	0.01	−0.05	−0.08	−0.13
Leaf width	−0.10	0.89**	0.09	0.08	−0.17	0.15	0.07	−0.18	0.10	−0.08	0.07	−0.02	−0.03
Petiole length	0.05	0.81**	−0.09	−0.02	−0.14	0.07	0.12	0.06	−0.33	0.12	0.00	0.04	0.24
Petiole width	0.01	0.45	−0.14	0.15	−0.36	−0.13	−0.14	−0.23	0.44	−0.22	−0.12	0.02	−0.30
Ripening date	−0.17	0.29	−0.10	0.02	0.01	0.61**	0.06	0.18	0.20	0.35	−0.02	−0.07	−0.18
Fruit density	−0.10	−0.07	−0.02	−0.04	−0.07	−0.04	−0.21	−0.15	−0.12	0.79**	0.00	−0.05	−0.12
Maximum of fruit number per node	−0.19	−0.25	0.19	−0.14	0.12	−0.11	0.11	0.77**	−0.18	−0.05	0.03	0.04	−0.20
Fruit stalk length	−0.02	−0.19	0.00	−0.09	−0.07	−0.26	0.03	0.03	0.78**	0.01	0.01	0.13	0.16
Fruit stalk diameter	0.16	−0.27	0.42	−0.31	0.36	0.03	0.34	−0.10	−0.34	−0.06	0.05	−0.07	−0.06
Fruit stalk length on bunch	−0.13	0.21	−0.25	−0.17	0.03	0.16	0.44	0.29	−0.06	0.05	0.01	0.59**	0.14
Fruit stalk diameter on bunch	−0.23	0.22	0.25	0.05	0.26	−0.17	0.26	−0.03	−0.04	0.44	0.30	−0.50	0.11
Fruit shape	−0.62**	−0.12	0.03	0.00	−0.14	0.04	−0.10	0.41	0.15	−0.19	−0.09	0.14	−0.39
Fruit length	0.71**	−0.06	0.35	0.07	0.36	−0.02	−0.09	0.24	0.12	−0.06	−0.17	0.07	−0.13
Fruit width	0.89**	0.06	0.22	0.03	0.22	0.01	−0.06	−0.15	−0.08	0.07	−0.01	−0.14	0.07
Fruit weight	0.81**	−0.20	0.22	−0.19	0.26	−0.11	0.16	0.14	−0.03	0.09	−0.07	0.02	0.05
Fruit color	−0.08	−0.08	−0.06	−0.18	−0.18	0.10	0.24	0.22	0.12	0.14	0.68**	0.25	0.33
Fruit flesh color	0.17	−0.25	0.05	−0.42	−0.12	0.12	0.50	−0.16	0.23	0.05	0.19	0.36	0.14
Fruit taste	−0.11	0.08	−0.04	0.13	0.17	−0.12	0.02	0.11	−0.05	0.01	0.83**	−0.05	−0.01
Fruit flesh firmness	0.05	−0.17	−0.77**	0.16	0.24	0.25	−0.05	−0.17	−0.01	−0.03	−0.05	0.01	0.14
Fruit flesh thickness	0.91**	0.08	−0.12	−0.04	−0.12	−0.15	−0.06	−0.08	0.04	−0.11	−0.10	−0.02	0.00
Fruit juice color	−0.65**	0.47	0.16	0.12	0.12	−0.29	0.07	−0.01	0.00	0.18	−0.08	−0.16	0.02
Seed number per fruit	0.08	−0.19	0.15	0.14	0.81**	0.14	0.06	0.12	−0.17	−0.01	0.09	0.00	−0.03
General shape of seeds	−0.01	0.22	−0.30	−0.22	−0.36	0.12	0.11	−0.02	0.51	−0.21	0.27	−0.04	0.24
Seed shape	0.21	−0.14	−0.03	−0.06	0.86**	−0.01	0.07	0.00	0.05	−0.09	0.05	0.06	0.00
Seed length	0.42	−0.04	0.70**	0.04	0.36	0.25	−0.11	0.10	−0.02	−0.03	−0.10	−0.07	−0.02
Seed width	0.29	0.11	0.71**	0.15	0.44	0.28	−0.08	−0.04	−0.10	0.04	−0.08	−0.01	0.08
Total weight of seeds	−0.09	0.02	0.52**	0.28	0.18	0.25	0.30	−0.09	0.51	0.07	0.05	−0.17	−0.03
Total	4.86	3.80	3.16	3.12	2.95	2.70	2.52	2.35	2.33	2.29	2.23	2.12	1.92
% of Variance	11.04	8.64	7.18	7.10	6.70	6.15	5.73	5.34	5.30	5.21	5.06	4.82	4.37
Cumulative %	11.04	19.68	26.86	33.96	40.66	46.80	52.54	57.88	63.18	68.38	73.45	78.27	82.64

**Eigenvalues ≥ 0.52 are significant at the *p* ≤ .01 level.

**FIGURE 3:**
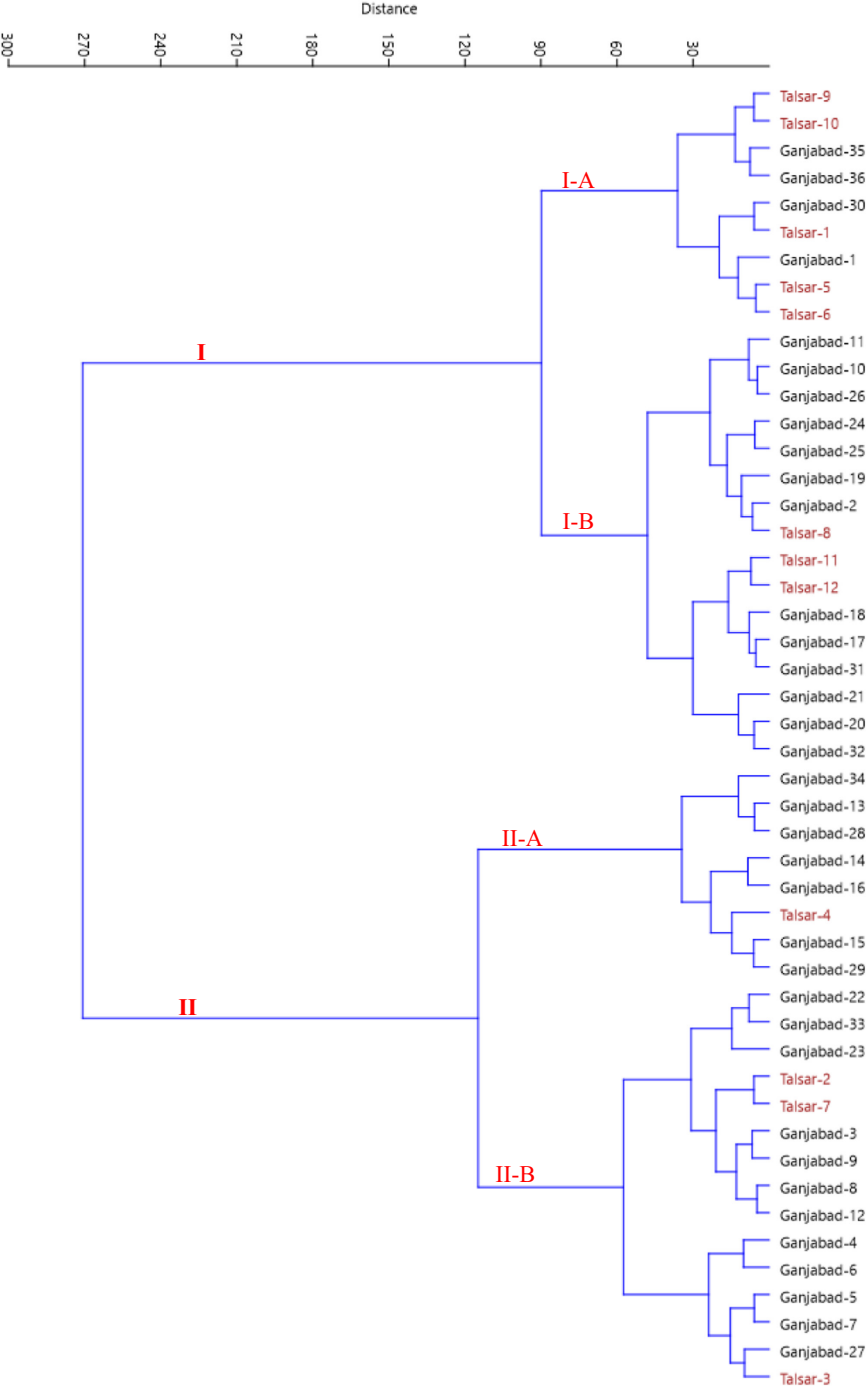
Ward cluster analysis of the studied *G. asiatica* accessions based on morphological traits using Euclidean distances

Also, the dendrogram created using cluster analysis grouped the accessions into two major clusters (Figure 3). The first cluster (I) was divided into two sub‐clusters. Sub‐cluster I‐A contained nine accessions, while 16 accessions formed sub‐cluster I‐B. Furthermore, the second cluster (II) was divided into two sub‐clusters. Sub‐cluster II‐A included eight accessions, while sub‐cluster II‐B consisted of 15 accessions. The derived clusters and sub‐clusters were similar to those identified from the bi‐plot.

Understanding the nature and amount of variability present in the genotypes of crops might be useful in improvement of traits of existing germplasm, which could be used in breeding programs of that crop. Furthermore, this kind of evaluation will provide information about uniqueness and distinctness of genotypes, which is of vital importance in optimal and effective conservation of genotypic variability (Dev et al., 2017).

## 4 CONCLUSION

Phalsa (*G. asiatica*) fruit has shown tremendous potential as a traditional, as well as functional food ingredient for formulating innovative beverages, and food preserves. Currently, the fruit is mostly used in traditional product formulation at cottage scale, or even at household scale. However, there is a huge potential for this important berry fruit to become an effective functional food with little efforts on improving its crop yield and developing a suitable cold supply chain for ensuring a safe supply to distant markets. Moreover, this berry fruit can also be effectively applied in various pharmaceutical applications, such as pain‐relief drugs, digestive aids, and drugs for controlling the glycemic index. Very short shelf life, seasonal availability, lack of awareness among people, and meager scientific literature are some major challenges associated with the commercial exploitation of phalsa fruit. Morphological attributes will provide important criteria for quality control of any fruit during sorting, grading handling, and processing operations. The present findings will be very helpful in the selection of best cultivars for further research work as well as to the consumption of fruit and food processing industries. Based on the traits related to fruit quality, such as fruit weight, fruit color, fruit flesh color, and fruit taste, 14 accessions, including Talsar‐6, Talsar‐8, Ganjabad‐31, Talsar‐4, Ganjabad‐18, Ganjabad‐24, Talsar‐5, Ganjabad‐25, Ganjabad‐30, Ganjabad‐17, Talsar‐7, Talsar‐3, Talsar‐2, and Talsar‐1, were superior. It is recommended to use the best accessions selected in breeding programs.

## FUNDING INFORMATION

None.

## CONFLICT OF INTEREST

The authors declare no conflict of interest.

## ETHICS STATEMENT

Research involving Human Participants and/or Animals: None.

## INFORMED CONSENT

None.

## Data Availability

The data that support the findings of this study are available from the corresponding author upon reasonable request.
